# Transgenerational Response of Germline Nuclear Hormone Receptor Genes to Nanoplastics at Predicted Environmental Doses in *Caenorhabditis elegans*

**DOI:** 10.3390/toxics12060420

**Published:** 2024-06-07

**Authors:** Zhengying Liu, Yuxing Wang, Qian Bian, Dayong Wang

**Affiliations:** 1 Key Laboratory of Environmental Medicine Engineering of Ministry of Education, Medical School, Southeast University, Nanjing 210009, China; 18326454044@163.com (Z.L.); w_yuxing1998@163.com (Y.W.); 2 Jiangsu Provincial Center for Disease Control and Prevention, Nanjing 210009, China; bianqian@jscdc.cn; 3 Shenzhen Ruipuxun Academy for Stem Cell & Regenerative Medicine, Shenzhen 518122, China

**Keywords:** nanoplastics, NHRs, transgenerational toxicity, nematode

## Abstract

Transgenerational nanoplastic toxicity could be detected in *Caenorhabditis elegans* after exposure at the parental generation (P0-G); however, the underlying mechanisms remain largely unclear. We aimed to examine the role of germline nuclear hormone receptors (NHRs) in controlling the transgenerational toxicity of polystyrene nanoparticles (PS-NPs) based on gene expression screening and functional analysis. Among germline NHR genes, *daf-12*, *nhr-14*, and *nhr-47* expressions were increased and *nhr-12* expression was decreased by PS-NPs (1 and 10 μg/L). Transgenerational alterations in expressions of these four NHR genes were also induced by PS-NPs (1 and 10 μg/L). RNAi of *daf-12*, *nhr-14*, and *nhr-47* caused resistance, whereas RNAi of *nhr-12* conferred susceptibility to transgenerational PS-NP toxicity. After PS-NP exposure, expressions of *ins-3*, *daf-28*, and *ins-39* encoding insulin ligands, *efn-3* encoding Ephrin ligand, and *lin-44* encoding Wnt ligand, as well as expressions of their receptor genes (*daf-2*, *vab-1*, and/or *mig-1*), were dysregulated by the RNAi of *daf-12*, *nhr-14*, *nhr-47*, and *nhr-12*. Therefore, alteration in certain germline NHRs could mediate the induction of transgenerational nanoplastic toxicity by affecting secreted ligands and their receptors in the offspring of exposed organisms.

## 1. Introduction

Due to improper handling, plastic pollution has been recognized as a global environmental hazard problem [1,2]. Moreover, because of insufficient degradation, microplastics and even nanoplastics are generated from waste plastics [3,4]. Nanoplastics have been widely distributed in different environments, such as water and soil [5,6], and can be transferred through environmental media [7,8]. Predicted environmental doses (PEDs) of nanoplastics range from ng/L to μg/L [9]. Nanoplastics were further detected and accumulated in the organs of several organisms, such as zebrafish [10,11]. Considering the existence of nanoplastics in terrestrial or aquatic food webs, their exposure risk for human health has been further implied [12,13]. Nanoplastics could even be discovered in human blood [14]. Once bioavailable to organisms, nanoplastics could be further internalized in cells and transported to certain organelles, such as mitochondria [15]. Accompanied with bioavailability and accumulation, some adverse effects on the development and functions of organs or tissues could be observed in different organisms after nanoplastic exposure [16,17,18,19]. Nanoplastic toxicity could be induced at both exposed parental generations (P0-G) and their offspring [20,21,22].

*Caenorhabditis elegans* exhibits high susceptibility to pollutant toxicity [23,24,25,26], which makes it suitable for assessing the toxicity of pollutants at PEDs [27,28,29]. Meanwhile, *C. elegans* has a relatively short life cycle [30], which makes it suitable to evaluate transgenerational toxicity after pollutant exposure [31,32]. In nematodes, transgenerational damage in gonads and neurons could be detected after exposure to pristine and aged nanoplastics [33,34]. For example, using locomotion behavior as the toxicity assessment endpoint, exposure to 10 μg/L of polystyrene nanoparticles (PS-NP) could cause the toxicity induction from P0-G to F4-G [35]. As the classic model animal, *C. elegans* can provide profound insights into the toxicological mechanisms of pollutants [36,37]. Some secreted germline ligands (such as insulin, FGF, and Wnt) and their receptors were shown to play an important function in governing transgenerational nanoplastic toxicity [38,39,40].

Some mechanisms (such as epigenetic regulation) exist to regulate the function of targets by affecting target expressions [41]. microRNAs and histone methylation regulation were identified to control transgenerational PS-NP toxicity [42,43,44]. As transcriptional factors, nuclear hormone receptors (NHRs) can respond to certain ligands to regulate some biological events by affecting target gene expression [45,46]. Among members in *C. elegans* [47], some NHRs (such as DAF-12) were proven to regulate the response to pollutants [48,49]. Environmental pollutants have been further implied to act as possible ligands to trigger or inhibit certain NHRs in nematodes after exposure [50,51]. We assumed that nanoplastics may induce transgenerational toxicity by activating or suppressing certain germline NHRs. Thus, in the current study, we aimed to identify germline NHRs involved in the regulation of transgenerational nanoplastic toxicity in nematodes. The PS-NP was selected as an example of nanoplastics. Moreover, the underlying mechanism for candidate germline NHRs in controlling the transgenerational PS-NP toxicity was further determined. Our results suggested the crucial function of germline NHRs in regulating the induction of transgenerational nanoplastic toxicity. The identified germline NHRs provide an important molecular basis for further elucidation of the molecular mechanisms for transgenerational nanoplastic toxicity in organisms.

## 2. Materials and Methods

### 2.1. Nanoplastic’s Properties

**Figure 1:**
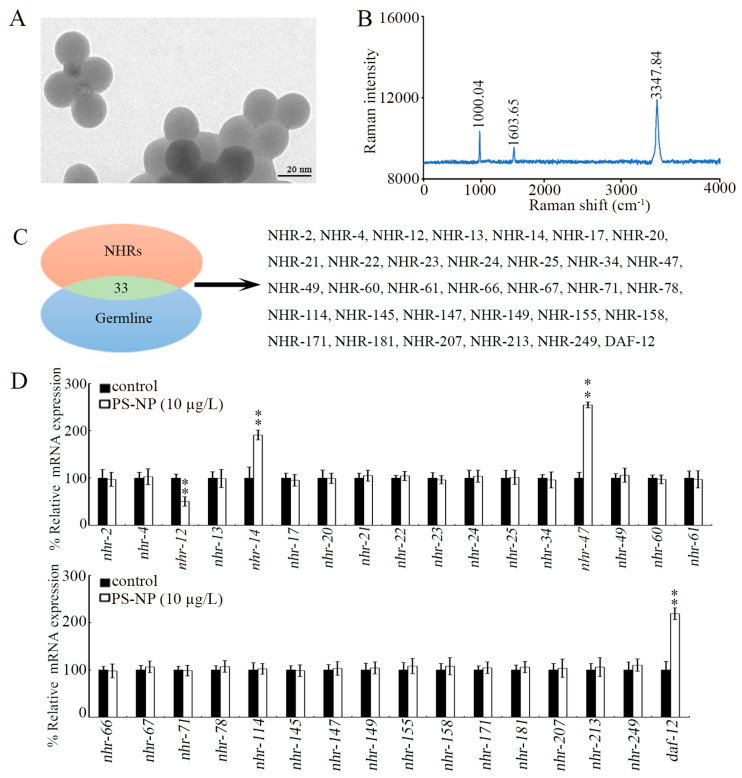
Effect of PS-NP exposure on expression of germline NHR genes. (**A**) TEM image of PS-NPs before sonication. (**B**) Raman spectrum of PS-NP. (**C**) NHR genes expressed in the germline. (**D**) Effect of PS-NP (10 μg/L) exposure on expressions of germline NHR genes. In total, 30 intact gonads were used for the qRT-PCR assay for each treatment. Data are presented as mean ± standard deviation (SD). ** *p <* 0.01 vs. control.

PS-NPs (20 nm) were purchased from Huge Biotechnol. Co. (Shanghai, China). Their morphology was spherical, and the particle size was 20.36 ± 1.8 nm, confirmed by transmission electron microscopy (TEM, FEI Talos F200X, Thermo Fisher Scientific, Waltham, MA, USA) (Figure 1A). Raman spectrum revealed two distinct peaks at 1603.65 cm^−1^ and 3347.84 cm^−1^, which correspond to symmetric carbon atom vibration in benzene rings, and at 1000.04 cm^−1^, indicating breathing benzene ring vibration (Figure 1B). Raman spectra were measured by a Raman Renishaw RM2000 (Renishaw, London, UK). The FTIR spectrum for PS-NPs was described by Liu et al. [35]. Based on FTIR spectrum analysis of PS-NPs, the peaks at 3082, 3059, and 3025 cm^−1^ were assigned to (=CH), the peaks at 2923 and 2849 cm^−1^ were assigned to (-CH_2_), the peaks at 1601, 1583, 1493, and 1452 cm^−1^ were assigned to (-C=C-), the peak at 1375 cm^−1^ was assigned to (-CH), and the peaks at 1065, 1028, 756, and 696 cm^−1^ were assigned to (=CH) [35].

### 2.2. Nematode Maintenance

As described by Brenner [52], animals (wild type, N2) were allowed to grow on nematode growth medium (NGM). *E. coli* OP50 was fed *C. elegans* as food. Both *E. coli* and all the nematodes were obtained from Caenorhabditis Genetics Center (CGC). The solution (2% HOCl, 0.45 M NaOH) was applied for synchronizing adults to collected eggs [53], which were then transferred onto new NGMs to grow into L1 larvae. Chemical reagents were purchased from Aladdin Industrial Corporation (La Puente, CA, USA) and Sangon Biotech Co., Ltd. (Shanghai, China).

### 2.3. Exposure

Concentrations (0.1–10 μg/L) of PS-NPs were used as described [54], which are the PEDs of nanoplastics [9]. The purchased PS-NPs were suspended in water. The working PS-NP suspensions were prepared by diluting the stocking solution with K buffer. To assess transgenerational PS-NP toxicity, *C. elegans* was exposed to PS-NPs from the L1 larval stage to adult day 3, referred to as P0-G. During the entire exposure period, PS-NP solutions were replaced daily to maintain consistent conditions. Eggs of P0-G were transferred to an NGM plate with OP50 added, allowing them to develop to the adulthood stage, which was called F1-G. The following offspring (F2-G to Fn-G) were generated successfully in this way. All the experiments were repeated three times. After the treatment, the animals were used simultaneously and separately for different research purposes. During the assessment of the endpoints for toxicity, animals were selected randomly.

### 2.4. Endpoints

To analyze reproductive capacity, the total number of offspring was defined as the brood size during the process of egg-laying [55]. To analyze locomotion, the frequency of body bend, as well as head thrash, was assessed [56]. After recovery on NGM for 1 min, these behaviors were examined under a stereomicroscope Nikon C-DSS230 stereomicroscope (Nikon, Japan) [57]. For each exposure, 50 *C. elegans* were examined. Three replicates were carried out.

### 2.5. Gene Expression

Trizol (Sangon Biotech Co., Ltd., Shanghai, China) was applied together with ceramic beads to grinder animals. Using M-MuLV reverse transcriptase (Sangon Biotech Co., Ltd., Shanghai, China), cDNAs were prepared. Quantitative real-time polymerase chain reaction (qRT-PCR) was carried out on a SYBR Green qRT-PCR master mix (Vazyme Biotech Co., Ltd., Nanjing, China). *tba-1* acted as the reference gene for the normalization of target genes [58]. To analyze the change in gene expression in the germline, intact gonads were separated and collected by removing them from the body of nematodes using a glass knife. Information on the primers is provided in Table S1. Three biological replicates were carried out.

### 2.6. RNA Interference (RNAi)

RNAi was generated through feeding with dsRNA-generating bacterial cells [59]. On an RNAi plate, L1 larval animals developed into adults. Their offspring were exposed to PS-NPs. L4440, an empty vector, functioned as the control [60]. DCL569 is a tool strain for the germline RNAi of genes. qRT-PCR was performed to analyze gene RNAi efficiency (Figure S1).

### 2.7. Data Analysis

To ensure the quality of data and avoid random errors, 50–100 nematodes were chosen randomly to measure the relative evaluation indexes. Meanwhile, three parallel experiments were performed simultaneously. Data were all continuous and passed the normality test and homogeneity test of variance in SPSS 26.0. Significant difference among treatments was analyzed by one-way or two-way ANOVA (for multi-factor comparison) followed by a post hoc test. A *p*-value of <0.01 (**) was considered as significant statistically.

## 3. Results

### 3.1. Identification of Germline NHRs in Response to PS-NP Exposure

In *C. elegans*, there are 33 NHR genes that can be expressed in the germline (Figure 1C). Among these 33 germline NHR genes, only 4 NHR genes could be dysregulated by exposure to PS-NPs (10 μg/L) (Figure 1D). The *nhr-12* expression was significantly decreased by exposure to 10 μg/L of PS-NPs, and the expressions of *nhr-14*, *nhr-47*, and *daf-12* were significantly increased by exposure to 10 μg/L of PS-NPs (Figure 1D).

### 3.2. Transgenerational Alteration in Expressions of Germline daf-12, nhr-12, nhr-14, and nhr-47 after PS-NP Exposure

**Figure 2:**
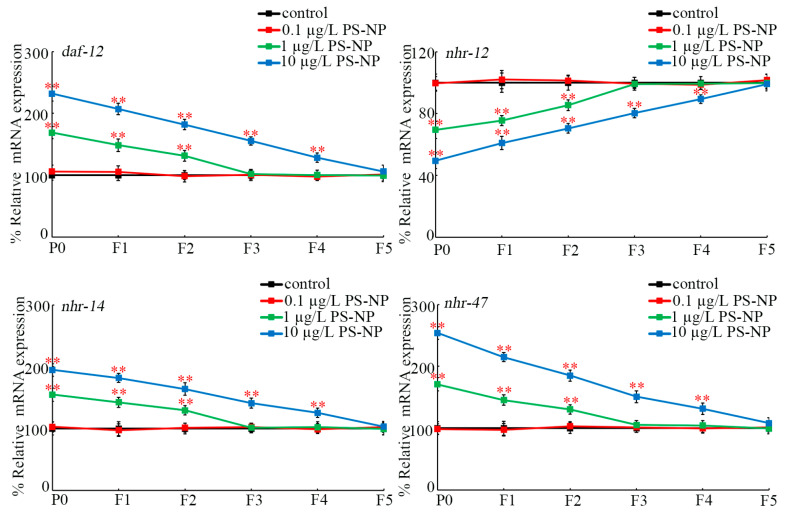
Transgenerational expressions of germline *daf-12*, *nhr-12*, *nhr-14*, and *nhr-47* after exposure to PS-NPs at P0-G. In total, 30 intact gonads were used for the qRT-PCR assay for each treatment. Data are presented as mean ± standard deviation (SD). ** *p <* 0.01 vs. control.

At P0-G, *daf-12*, *nhr-14*, and *nhr-47* expressions were increased and *nhr-12* expression was decreased by exposure to 1 and 10 μg/L of PS-NPs, whereas expressions of these four genes were not affected by exposure to 0.1 μg/L of PS-NPs (Figure 2). Moreover, exposure to 1 μg/L of PS-NPs caused increased *daf-12*, *nhr-14*, and *nhr-47* expressions and decreased *nhr-12* expression at F1-G and at F2-G compared to the control (Figure 2). Additionally, exposure to 10 μg/L of PS-NPs resulted in increased *daf-12*, *nhr-14*, and *nhr-47* expressions and decreased *nhr-12* expression from F1-G to F4-G compared to the control (Figure 2).

### 3.3. RNAi of daf-12, nhr-12, nhr-14, and nhr-47 Affected Transgenerational PS-NP Toxicity

**Figure 3:**
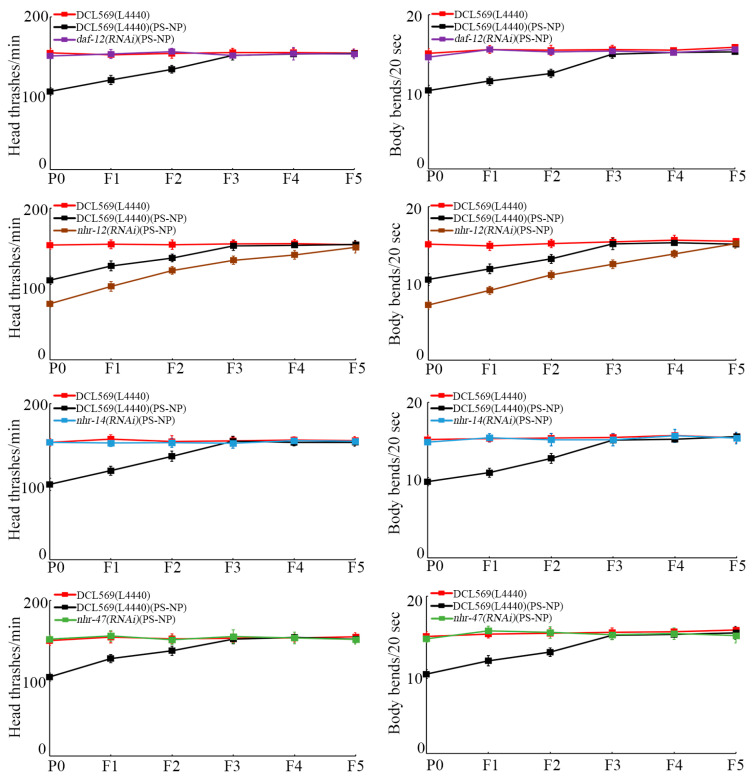
Effect of RNAi of *daf-12*, *nhr-12*, *nhr-14*, and *nhr-47* on transgenerational PS-NP toxicity in decreasing locomotion behavior. Exposure concentration of PS-NPs was 1 μg/L. The curves of DCL569(L4440)(PS-NP) were significantly (*p* < 0.01) different from those of DCL569(L4440). The curves of *daf-12(RNAi)*(PS-NP), *nhr-12(RNAi)*(PS-NP), *nhr-14(RNAi)*(PS-NP), and *nhr-47(RNAi)*(PS-NP) were significantly (*p* < 0.01) different from those of DCL569(L4440)(PS-NP). Significance between curves was tested by Kaplan–Meier analysis. Data are presented as mean ± standard deviation (SD).

With locomotion behavior as the endpoint to reflect the function of motor neurons, the transgenerational PS-NP toxicity to decrease locomotion behavior was inhibited by germline RNAi of *daf-12*, *nhr-14*, and *nhr-47* (Figure 3). Different from this, the transgenerational PS-NP toxicity to decrease locomotion behavior could be strengthened by germline RNAi of *nhr-12* (Figure 3).

**Figure 4:**
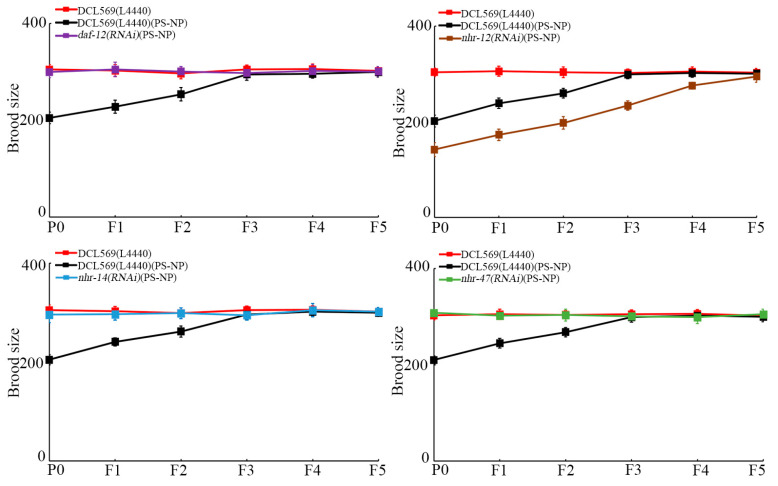
Effect of RNAi of *daf-12*, *nhr-12*, *nhr-14*, and *nhr-47* on transgenerational PS-NP toxicity in reducing brood size. Exposure concentration of PS-NPs was 1 μg/L. The curves of DCL569(L4440)(PS-NP) were significantly (*p* < 0.01) different from those of DCL569(L4440). The curves of *daf-12(RNAi)*(PS-NP), *nhr-12(RNAi)*(PS-NP), *nhr-14(RNAi)*(PS-NP), or *nhr-47(RNAi)*(PS-NP) were significantly (*p* < 0.01) different from those of DCL569(L4440)(PS-NP). Significance between curves was tested by Kaplan–Meier analysis. Data are presented as mean ± standard deviation (SD).

With brood size as the endpoint to reflect reproductive capacity, similarly, germline RNAi of *daf-12*, *nhr-14*, and *nhr-47* further suppressed the transgenerational PS-NP toxicity to reduce brood size, and germline RNAi of *nhr-12* increased the transgenerational PS-NP toxicity to reduce brood size (Figure 4). Therefore, *daf-12(RNAi)*, *nhr-14(RNAi)*, and *nhr-47(RNAi)* nematodes showed resistance to transgenerational PS-NP toxicity, whereas *nhr-12(RNAi)* nematodes exhibited susceptibility to transgenerational PS-NP toxicity.

### 3.4. Germline RNAi of daf-12, nhr-12, nhr-14, and nhr-47 Affected Expressions of Certain Secreted Ligand Genes in PS-NP-Exposed Nematodes

**Figure 5:**
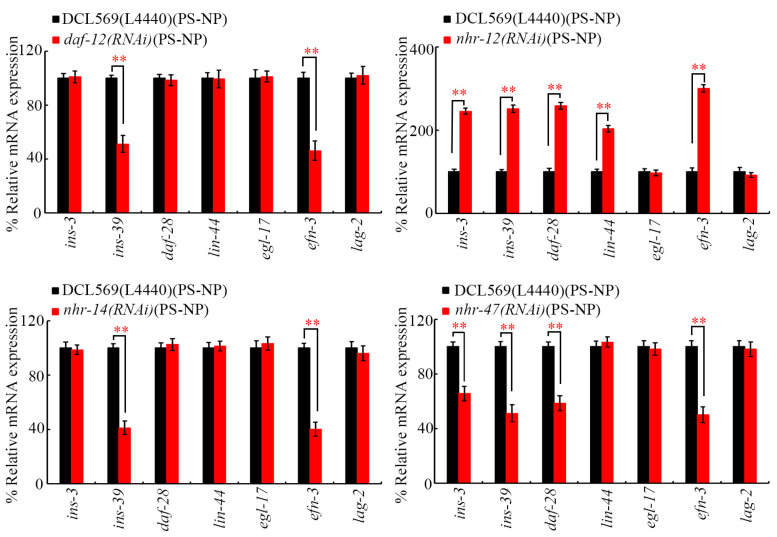
Effect of germline RNAi of *daf-12*, *nhr-12*, *nhr-14*, and *nhr-47* on expressions of secreted ligand genes in PS-NP-exposed nematodes. Exposure concentration of PS-NPs was 1 μg/L. In total, 30 intact gonads were used for the qRT-PCR assay for each treatment. Data are presented as mean ± standard deviation (SD). ** *p <* 0.01.

Under PS-NP exposure conditions, the RNAi of *daf-12* and *nhr-14* decreased expressions of germline *ins-39* and *efn-3* (Figure 5). In PS-NP-exposed nematodes, the RNAi of *nhr-12* increased expressions of germline *ins-3*, *ins-39*, *daf-28*, *lin-44*, and *efn-3* (Figure 5). Additionally, the RNAi of *nhr-47* decreased expressions of germline *ins-3*, *ins-39*, *daf-28*, and *efn-3* in PS-NP-exposed animals (Figure 5). In contrast, in PS-NP-exposed nematodes, expressions of *daf-28*, *lin-44*, and *egl-17* were not altered by the RNAi of *daf-12* and *nhr-14*, expressions of *egl-17* and *lag-2* were not affected by the RNAi of *nhr-12*, and expressions of *lin-44*, *egl-17*, and *lag-2* were not changed by the RNAi of *nhr-47* (Figure 5). INS-3, INS-39, and DAF-28 are insulin ligands, LIN-44 is a Wnt ligand, EGL-17 is an FGF ligand, EFN-3 is an Ephrin ligand, and LAG-2 is a Notch ligand [38,39,40,61,62].

### 3.5. Germline RNAi of daf-12, nhr-12, nhr-14, and nhr-47 Altered Expressions of Receptor Genes of Corresponding Insulin, Ephrin, and Wnt Ligand Genes in the Offspring of PS-NP-Exposed Nematodes

**Figure 6:**
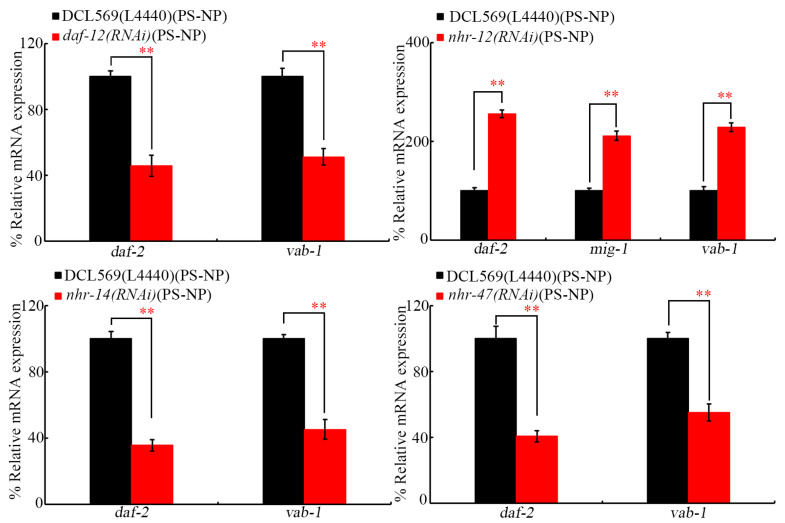
Effect of germline RNAi of *daf-12*, *nhr-12*, *nhr-14*, and *nhr-47* on expressions of *daf-2*, *vab-1*, and/or *mig-1* at F1-G of PS-NP-exposed nematodes. Exposure concentration of PS-NPs was 1 μg/L. Data are presented as mean ± standard deviation (SD). ** *p <* 0.01.

After PS-NP exposure at P0-G, the RNAi of *daf-12*, *nhr-14*, and *nhr-47* further decreased *daf-2* and *vab-1* expressions (Figure 6). In addition, after PS-NP exposure at P0-G, the expressions of *daf-2*, *mig-1*, and *vab-1* were increased by RNAi of *nhr-12* at F1-G (Figure 6). DAF-2 is an insulin receptor, VAB-1 is an Ephrin receptor, and MIG-1 is a Wnt receptor [30,31,53].

## 4. Discussion

Increasing evidence has indicated the important involvement of NHRs in governing stress response [63,64]. In addition, NHRs were shown to have a function in response to pollutant exposure in organisms at P0-G [65,66]. Some intestinal NHRs (such as NHR-8 and DAF-12) were identified to function in regulating nanoplastic toxicity in nematodes at P0-G [49,50]. For example, intestinal NHR-8 regulated PS-NP toxicity by activating DAF-12 [50]. In the TGF-β signaling pathway, intestinal DAF-3 and DAF-5 regulated nanoplastic toxicity by inhibiting DAF-12 function [49]. In contrast, little is known about the role of germline NHRs in response to pollutants, including nanoplastics. In the current study, among germline NHR genes, expressions of only four germline NHR genes were dysregulated by exposure to 10 μg/L of PS-NPs (Figure 1D). The 10 μg/L concentration is a PED for nanoplastics [9]. This suggested that a limited number of germline NHRs could exhibit the response to nanoplastics at PEDs at P0-G. After PS-NP (10 μg/L) exposure, we observed increased germline *nhr-14*, *nhr-47*, and *daf-12* expressions and decreased germline *nhr-12* expression (Figure 1D). These suggested that, in the germline, NHR-12 may exhibit a different function from NHR-14, NHR-47, and DAF-12 in controlling nanoplastic toxicity. In *C. elegans*, germline NHR-14 regulated DNA damage [67] and acted together with DNA damage checkpoints to control nanoplastic reproductive toxicity [68]. In addition, inhibition in germ proliferation by dafachronic acid was DAF-12-dependent [69].

In addition to the response to PS-NP at P0-G, we further observed the response of germline NHR genes in the offspring. Increased germline *daf-12*, *nhr-14*, and *nhr-47* expressions and decreased *nhr-12* expression were detected from P0-G to F2-G of PS-NP (1 μg/L)-exposed nematodes and from P0-G to F4-G of PS-NP (10 μg/L)-exposed nematodes (Figure 2). Thus, after nanoplastic exposure at PEDs at P0-G, these four germline NHR genes could exhibit a response across multiple generations. Additionally, the effect of these four germline NHR genes was not restricted at P0-G, and they would exert their effect on the offspring of PS-NP-exposed nematodes once activated or inhibited by PS-NPs at P0-G.

Moreover, using locomotion behavior and reproduction as endpoints, we detected resistance of *daf-12(RNAi)*, *nhr-14(RNAi)*, and *nhr-47(RNAi)* nematodes and susceptibility of *nhr-12(RNAi)* to transgenerational PS-NP toxicity (Figure 3 and Figure 4). At P0-G, the resistance of *daf-12(RNAi)* and *nhr-14(RNAi)* nematodes to nanoplastic toxicity was observed previously [40,59]. These findings demonstrated that, in the germline, the transgenerational activation of DAF-12, NHR-14, and NHR-47 and transgenerational suppression in NHR-12 mediated PS-NP toxicity formation across multiple generations. That is, the transgenerational response of these four germline NHRs functioned as a crucial contributor to the induction of transgenerational nanoplastic toxicity in nematodes. Nevertheless, after P0-G PS-NP (10 μg/L) exposure, activation of DAF-12, NHR-14, and NHR-47 and suppression in NHR-12 were recovered to control levels at F5-G (Figure 2). This implied that some unidentified signals exist to inhibit or block the further transgenerational germline NHR response caused by P0-G PS-NP exposure. The specificity of the RNAi effect to recover locomotive behavior and reproductive capacity using selected NHR genes in the germline (i.e., *daf-12*, *nhr-12*, *nhr-14*, and *nhr-47*) was not evaluated using other NHR genes whose expression was unaffected by exposure to PS-NP (Figure 1). Therefore, we cannot exclude the possibility that RNAi of some of them may also affect transgenerational PS-NP toxicity.

In nematodes, germline insulin, Wnt, FGF, Ephrin, and Notch ligands controlled transgenerational nanoplastic toxicity [38,39,40,61,62]. In PS-NP-exposed nematodes, we further found that germline *ins-39* and *efn-3* expressions were inhibited by RNAi of *daf-12* and *nhr-14*, germline *ins-3*, *ins-39*, *daf-28*, *lin-44*, and *efn-3* expressions were increased by RNAi of *nhr-12*, and germline *ins-3*, *ins-39*, *daf-28*, and *efn-3* expressions were inhibited by RNAi of *nhr-47* (Figure 5). Moreover, at F1-G of PS-NP-exposed nematodes, *daf-2* and *vab-1* expressions were decreased by RNAi of *daf-12*, *nhr-14*, and *nhr-47* and increased by RNAi of *nhr-12* RNAi, and *mig-1* expression was also increased by RNAi of *nhr-12* (Figure 6). In nematodes, germline RNAi of *ins-3*, *ins-39*, *daf-28*, *lin-44*, and *efn-3* conferred resistance to transgenerational PS-NP toxicity [39,40,62]. These observations provided an important molecular basis for these four germline NHRs in controlling transgenerational nanoplastic toxicity. These results further confirmed the role of alterations in these four germline NHRs in mediating the formation of transgenerational PS-NP toxicity.

In *C. elegans*, insulin receptor DAF-2, Ephrin receptor VAB-1, and Wnt receptor MIG-1 have been proven to regulate nanoplastic toxicity [39,40,62]. RNAi of *daf-2*, *vab-1*, and *mig-1* further induced resistance to transgenerational PS-NP toxicity [39,40,62]. Additionally, these four germline NHRs control transgenerational PS-NP toxicity by differentially targeting insulin, Wnt, and/or Ephrin ligands to a certain degree, which further affects the function of their receptors in their offspring. Nevertheless, the expression of germline *egl-17* and *lag-2* was not affected by RNAi of these four NHR genes under PS-NP exposure conditions (Figure 5). The RNAi of *egl-17* and *lag-2* also caused resistance to transgenerational PS-NP toxicity [38,61]. This implies that certain unidentified upstream regulators exist to activate or inhibit FGF and Notch ligands that mediate transgenerational nanoplastic toxicity.

## 5. Conclusions

Together, using *C. elegans* as an animal model, we identified four germline NHR genes (*daf-12*, *nhr-12*, *nhr-14*, and *nhr-47*) with a transgenerational response to exposure to PS-NPs at PEDs (1 and 10 μg/L). After PS-NP exposure at P0-G, expressions of germline *daf-12*, *nhr-14*, and *nhr-47* exhibited a transgenerational increase, and expression of germline *nhr-12* showed a transgenerational decrease. In the germline, NHR-12 and DAF-12, NHR-14, or NHR-47 had opposite functions during controlling transgenerational PS-NP toxicity in decreasing locomotion behavior and in reducing brood size. Moreover, DAF-12, NHR-12, NHR-14, and NHR-47 regulated transgenerational PS-NP toxicity by affecting the expressions of certain secreted ligands (INS-3, INS-39, DAF-28, LIN-44, and EFN-3) and their receptors (DAF-2, MIG-1, and VAB-1) in the offspring. Our results highlight the crucial role of alteration in germline NHRs in mediating transgenerational toxicity of nanoplastics at PEDs in organisms. Further high-throughput screening of downstream germline targets for NHR-12, DAF-12, NHR-14, and NHR-47 will provide a deeper understanding of the molecular mechanism of transgenerational toxicity induction of nanoplastics.

## Data Availability

The data presented in this study are available on request from the corresponding author.

## Supplementary Materials

The following supporting information can be downloaded at https://www.mdpi.com/article/10.3390/toxics12060420/s1, Figure S1: RNAi efficiency of *daf-12*, *nhr-12*, *nhr-14* and *nhr-47*; Table S1: Primer information for qRT-PCR.

## References

[1] MacLeod M., Arp H.P.H., Tekman M.B., Jahnke A. (2021). The global threat from plastic pollution. Science.

[2] Bouchet V.M.P., Seuront L., Tsujimoto A., Richirt J., Frontalini F., Tsuchiya M., Matsuba M., Nomaki H. (2023). Foraminifera and plastic pollution: Knowledge gaps and research opportunities. Environ. Pollut..

[3] Jeong J., Im J., Choi J. (2024). Integrating aggregate exposure pathway and adverse outcome pathway for micro/nanoplastics: A review on exposure, toxicokinetics, and toxicity studies. Ecotoxicol. Environ. Saf..

[4] Shukla S., Pei Y., Li W.G., Pei D.S. (2024). Toxicological research on nano and microplastics in environmental pollution: Current advances and future directions. Aquat. Toxicol..

[5] Trevisan R., Ranasinghe P., Jayasundara N., Di Giulio R.T. (2022). Nanoplastics in aquatic environments: Impacts on aquatic species and interactions with environmental factors and pollutants. Toxics.

[6] Leistenschneider D., Wolinski A., Cheng J., Ter Halle A., Duflos G., Huvet A., Paul-Pont I., Lartaud F., Galgani F., Lavergne É. (2023). A critical review on the evaluation of toxicity and ecological risk assessment of plastics in the marine environment. Sci. Total Environ..

[7] Huang D., Chen H., Shen M., Tao J., Chen S., Yin L., Zhou W., Wang X., Xiao R., Li R. (2022). Recent advances on the transport of microplastics/nanoplastics in abiotic and biotic compartments. J. Hazard. Mater..

[8] Pradel A., Catrouillet C., Gigault J. (2023). The environmental fate of nanoplastics: What we know and what we need to know about aggregation. NanoImpact.

[9] Lenz R., Enders K., Nielsen T.G. (2016). Microplastic exposure studies should be environmentally realistic. Proc. Natl. Acad. Sci. USA.

[10] Niu H., Liu S., Jiang Y., Hu Y., Li Y., He L., Xing M., Li X., Wu L., Chen Z. (2023). Are microplastics toxic? A review from eco-toxicity to effects on the gut microbiota. Metabolites.

[11] Li Y., Xia X., Zhang J., Lin X., Zhang Y., Wang H., Li Y., Zhang Q., Zhang S. (2023). Bioavailability of micro/nanoplastics and their associated polycyclic aromatic hydrocarbons to *Daphnia Magna*: Role of ingestion and egestion of plastics. Sci. Total Environ..

[12] Wang W., Do A.T.N., Kwon J.H. (2022). Ecotoxicological effects of micro- and nanoplastics on terrestrial food web from plants to human beings. Sci. Total Environ..

[13] Feng Y., Tu C., Li R., Wu D., Yang J., Xia Y., Peijnenburg W.J.G.M., Luo Y. (2023). A systematic review of the impacts of exposure to micro- and nano-plastics on human tissue accumulation and health. Eco. Environ. Health.

[14] Leslie H.A., van Velzen M.J.M., Brandsma S.H., Vethaak A.D., Garcia-Vallejo J.J., Lamoree M.H. (2022). Discovery and quantification of plastic particle pollution in human blood. Environ. Int..

[15] Hua X., Wang D.-Y. (2022). Cellular uptake, transport, and organelle response after exposure to microplastics and nanoplastics: Current knowledge and perspectives for environmental and health risks. Rev. Environ. Contam. Toxicol..

[16] Hirt N., Body-Malapel M. (2020). Immunotoxicity and intestinal effects of nano- and microplastics: A review of the literature. Part. Fibre Toxicol..

[17] Ye J., Ren Y., Dong Y., Fan D. (2024). Understanding the impact of nanoplastics on reproductive health: Exposure pathways, mechanisms, and implications. Toxicology.

[18] Tang M., Ding G., Lu X., Huang Q., Du H., Xiao G., Wang D. (2022). Exposure to nanoplastic particles enhances *Acinetobacter* survival, biofilm formation, and serum resistance. Nanomaterials.

[19] Tang M., Ding G., Li L., Xiao G., Wang D. (2023). Exposure to polystyrene nanoparticles at predicted environmental concentrations enhances toxic effects of *Acinetobacter johnsonii* AC15 infection on *Caenorhabditis elegans*. Ecotoxicol. Environ. Saf..

[20] Junaid M., Liu S., Chen G., Liao H., Wang J. (2023). Transgenerational impacts of micro(nano)plastics in the aquatic and terrestrial environment. J. Hazard. Mater..

[21] Yi J., Ma Y., Ruan J., You S., Ma J., Yu H., Zhao J., Zhang K., Yang Q., Jin L. (2024). The invisible Threat: Assessing the reproductive and transgenerational impacts of micro- and nanoplastics on fish. Environ. Int..

[22] Zhuang Z., Liu T., Liu Z., Wang D. (2024). Polystyrene nanoparticles strengthen high glucose toxicity associated with alteration in insulin signaling pathway in *C. elegans*. Ecotoxicol. Environ. Saf..

[23] Wang D.-Y. (2020). Exposure Toxicology in Caenorhabditis elegans.

[24] Long N.P., Kang J.S., Kim H.M. (2023). *Caenorhabditis elegans*: A model organism in the toxicity assessment of environmental pollutants. Environ. Sci. Pollut. Res. Int..

[25] Shao Y., Wang Y., Hua X., Li Y., Wang D. (2023). Polylactic acid microparticles in the range of μg/L reduce reproductive capacity by affecting the gonad development and the germline apoptosis in *Caenorhabditis elegans*. Chemosphere.

[26] Shao Y., Hua X., Li Y., Wang D. (2024). Comparison of reproductive toxicity between pristine and aged polylactic acid microplastics in *Caenorhabditis elegans*. J. Hazard. Mater..

[27] Wang Y.-X., Hua X., Wang D.-Y. (2023). Exposure to 6-PPD quinone enhances lipid accumulation through activating metabolic sensors of SBP-1 and MDT-15 in *Caenorhabditis elegans*. Environ. Pollut..

[28] Wang Y.-X., Liang G.-Y., Chao J., Wang D.-Y. (2024). Comparison of intestinal toxicity in enhancing intestinal permeability and in causing ROS production of six PPD quinones in *Caenorhabditis elegans*. Sci. Total Environ..

[29] Hua X., Wang D.-Y. (2023). Disruption of dopamine metabolism by exposure to 6-PPD quinone in *Caenorhabditis elegans*. Environ. Pollut..

[30] Zhao Y., Chen J., Wang R., Pu X., Wang D. (2023). A review of transgenerational and multigenerational toxicology in the in vivo model animal *Caenorhabditis elegans*. J. Appl. Toxicol..

[31] Chowdhury M.I., Sana T., Panneerselvan L., Sivaram A.K., Megharaj M. (2022). Perfluorooctane sulfonate (PFOS) induces several behavioural defects in *Caenorhabditis elegans* that can also be transferred to the next generations. Chemosphere.

[32] Li H., Zeng L., Wang C., Shi C., Li Y., Peng Y., Chen H., Zhang J., Cheng B., Chen C. (2022). Review of the toxicity and potential molecular mechanisms of parental or successive exposure to environmental pollutants in the model organism *Caenorhabditis elegans*. Environ. Pollut..

[33] Chen H., Gu Y., Jiang Y., Yu J., Chen C., Shi C., Li H. (2023). Photoaged polystyrene nanoplastics result in transgenerational reproductive toxicity associated with the methylation of histone H3K4 and H3K9 in *Caenorhabditis elegans*. Environ. Sci. Technol..

[34] Liu H., Wu Y., Wang Z. (2023). Long-term exposure to polystyrene nanoparticles at environmentally relevant concentration causes suppression in heme homeostasis signal associated with transgenerational toxicity induction in *Caenorhabditis elegans*. J. Hazard. Mater..

[35] Liu H.-L., Tian L.-J., Wang S.-T., Wang D.-Y. (2021). Size-dependent transgenerational toxicity induced by nanoplastics in nematode *Caenorhabditis elegans*. Sci. Total Environ..

[36] von Mikecz A. (2022). Exposome, molecular pathways and one health: The invertebrate *Caenorhabditis elegans*. Int. J. Mol. Sci..

[37] Hua X., Liang G.-Y., Chao J., Wang D.-Y. (2024). Exposure to 6-PPD quinone causes damage on mitochondrial complex I/II associated with lifespan reduction in *Caenorhabditis elegans*. J. Hazard. Mater..

[38] Hua X., Cao C., Zhang L., Wang D. (2023). Activation of FGF signal in germline mediates transgenerational toxicity of polystyrene nanoparticles at predicted environmental concentrations in *Caenorhabditis elegans*. J. Hazard. Mater..

[39] Xu R., Hua X., Rui Q., Wang D. (2022). Alteration in Wnt signaling mediates induction of transgenerational toxicity of polystyrene nanoplastics in *C. elegans*. NanoImpact.

[40] Liu H., Zhao Y., Hua X., Wang D. (2022). Induction of transgenerational toxicity is associated with the activated germline insulin signals in nematodes exposed to nanoplastic at predicted environmental concentrations. Ecotoxicol. Environ. Saf..

[41] Gleason R.J., Chen X. (2023). Epigenetic dynamics during germline development: Insights from *Drosophila* and *C. elegans*. Curr. Opin. Genet. Dev..

[42] Zhang L., Wang S.-T., Zhao Y., Bi K., Wang D.-Y. (2022). Increase in germline methyltransferases governing methylation of histone H3K9 is associated with transgenerational nanoplastic toxicity in *Caenorhabditis elegans*. Environ. Sci. Nano.

[43] Hua X., Zhao Y., Yuan Y.-J., Zhang L., Bian Q., Wang D.-Y. (2022). Nanoplastics cause transgenerational toxicity through inhibiting germline microRNA *mir-38* in *C. elegans*. J. Hazard. Mater..

[44] Yu C.W., Luk T.C., Liao V.H. (2021). Long-term nanoplastics exposure results in multi and trans-generational reproduction decline associated with germline toxicity and epigenetic regulation in *Caenorhabditis elegans*. J. Hazard. Mater..

[45] Taubert S., Ward J.D., Yamamoto K.R. (2011). Nuclear hormone receptors in nematodes: Evolution and function. Mol. Cell. Endocrinol..

[46] Tao L.J., Seo D.E., Jackson B., Ivanova N.B., Santori F.R. (2020). Nuclear Hormone Receptors and Their Ligands: Metabolites in Control of Transcription. Cells.

[47] Antebi A. (2015). Nuclear Receptor Signal Transduction in *C. elegans*. WormBook.

[48] Shomer N., Kadhim A.Z., Grants J.M., Cheng X., Alhusari D., Bhanshali F., Poon A.F., Lee M.Y.Y., Muhuri A., Park J.I. (2019). Mediator subunit MDT-15/MED15 and nuclear receptor HIZR-1/HNF4 cooperate to regulate toxic metal stress responses in *Caenorhabditis elegans*. PLoS Genet..

[49] Wang S., Liu H., Zhao Y., Rui Q., Wang D. (2020). Dysregulated *mir-354* enhanced the protective response to nanopolystyrene by affecting the activity of TGF-β signaling pathway in nematode *Caenorhabditis elegans*. NanoImpact.

[50] Liu H., Shao H., Guo Z., Wang D. (2020). Nanopolystyrene exposure activates a fat metabolism related signaling-mediated protective response in *Caenorhabditis elegans*. NanoImpact.

[51] Hartman J.H., Widmayer S.J., Bergemann C.M., King D.E., Morton K.S., Romersi R.F., Jameson L.E., Leung M.C.K., Andersen E.C., Taubert S. (2021). Xenobiotic metabolism and transport in *Caenorhabditis elegans*. J. Toxicol. Environ. Health B Crit. Rev..

[52] Brenner S. (1974). The genetics of *Caenorhabditis elegans*. Genetics.

[53] Wang Y.-X., Zhang L., Yuan X.-A., Wang D.-Y. (2023). Treatment with paeoniflorin increases lifespan of *Pseudomonas aeruginosa* infected *Caenorhabditis elegans* by inhibiting bacterial accumulation in intestinal lumen and biofilm formation. Front. Pharmacol..

[54] Xu R., Hua X., Rui Q., Wang D. (2022). Polystyrene nanoparticles caused dynamic alteration in mitochondrial unfolded protein response from parents to the offspring in *C. elegans*. Chemosphere.

[55] Liu Z.-Y., Bian Q., Wang D.-Y. (2024). Exposure to 6-PPD quinone causes ferroptosis activation associated with induction of reproductive toxicity in *Caenorhabditis elegans*. J. Hazard. Mater..

[56] Hua X., Wang D.-Y. (2023). Exposure to 6-PPD quinone at environmentally relevant concentrations inhibits both lifespan and healthspan in *C. elegans*. Environ. Sci. Technol..

[57] Hua X., Wang D.-Y. (2024). Polyethylene nanoparticles at environmentally relevant concentrations enhances neurotoxicity and accumulation of 6-PPD quinone in *Caenorhabditis elegans*. Sci. Total Environ..

[58] Yuan C., Wang Y., Zhang L., Wang D. (2024). Procatechuic acid and protocatechuic aldehyde increase survival of *Caenorhabditis elegans* after fungal infection and inhibit fungal virulence. Front. Pharmacol..

[59] Zhang L., Wang Y.-X., Wang D.-Y. (2023). Paeoniflorin increases the survival of *Pseudomonas aeruginosa* infected *Caenorhabditis elegans* at the immunosuppression stage by activating PMK-1, BAR-1, and EGL-1 signals. Arch. Pharm. Res..

[60] Liu T., Zhuang Z., Wang D. (2023). Paeoniflorin mitigates high glucose-induced lifespan reduction by inhibiting insulin signaling in *Caenorhabditis elegans*. Front. Pharmacol..

[61] He W., Gu A., Wang D. (2023). Sulfonate-modified polystyrene nanoparticle at precited environmental concentrations induces transgenerational toxicity associated with increase in germline Notch signal of *Caenorhabditis elegans*. Toxics.

[62] Zhao Y., Hua X., Bian Q., Wang D.-Y. (2022). Nanoplastic exposure at predicted environmental concentrations induces activation of germline Ephrin signal associated with toxicity formation in the *Caenorhabditis elegans* offspring. Toxics.

[63] Ma L., Nelson E.R. (2019). Oxysterols and nuclear receptors. Mol. Cell. Endocrinol..

[64] Doering K.R.S., Ermakova G., Taubert S. (2023). Nuclear hormone receptor NHR-49 is an essential regulator of stress resilience and healthy aging in *Caenorhabditis elegans*. Front. Physiol..

[65] Gustafsson J.A. (1995). Receptor-mediated toxicity. Toxicol. Lett..

[66] Abe T., Takahashi M., Kano M., Amaike Y., Ishii C., Maeda K., Kudoh Y., Morishita T., Hosaka T., Sasaki T. (2017). Activation of nuclear receptor CAR by an environmental pollutant perfluorooctanoic acid. Arch. Toxicol..

[67] Sang L., Dong R., Liu R., Hao Q., Bai W., Sun J. (2022). *Caenorhabditis elegans* NHR-14/HNF4alpha regulates DNA damage-induced apoptosis through cooperating with *cep-1*/p53. Cell Commun. Signal.

[68] Liu Z.-Y., Hua X., Zhao Y., Bian Q., Wang D.-Y. (2024). Polyethylene nanoplastics cause reproductive toxicity associated with activation of both estrogenic hormone receptor NHR-14 and DNA damage checkpoints in *C. elegans*. Sci. Total Environ..

[69] Mukherjee M., Chaudhari S.N., Balachandran R.S., Vagasi A.S., Kipreos E.T. (2017). Dafachronic acid inhibits *C. elegans* germ cell proliferation in a DAF-12-dependent manner. Dev. Biol..

