# Supplementary material for: Transgenerational Response of Germline Nuclear Hormone Receptor Genes to Nanoplastics at Predicted Environmental Doses in Caenorhabditis elegans
Source: Toxics. 2024 Jun 7;12(6):420. doi: 10.3390/toxics12060420 (PMC11209457; doi:10.3390/toxics12060420)
Supplement: Supplementary file 1 [file toxics-12-00420-s001.zip › toxics-3035859-supplementary.pdf]

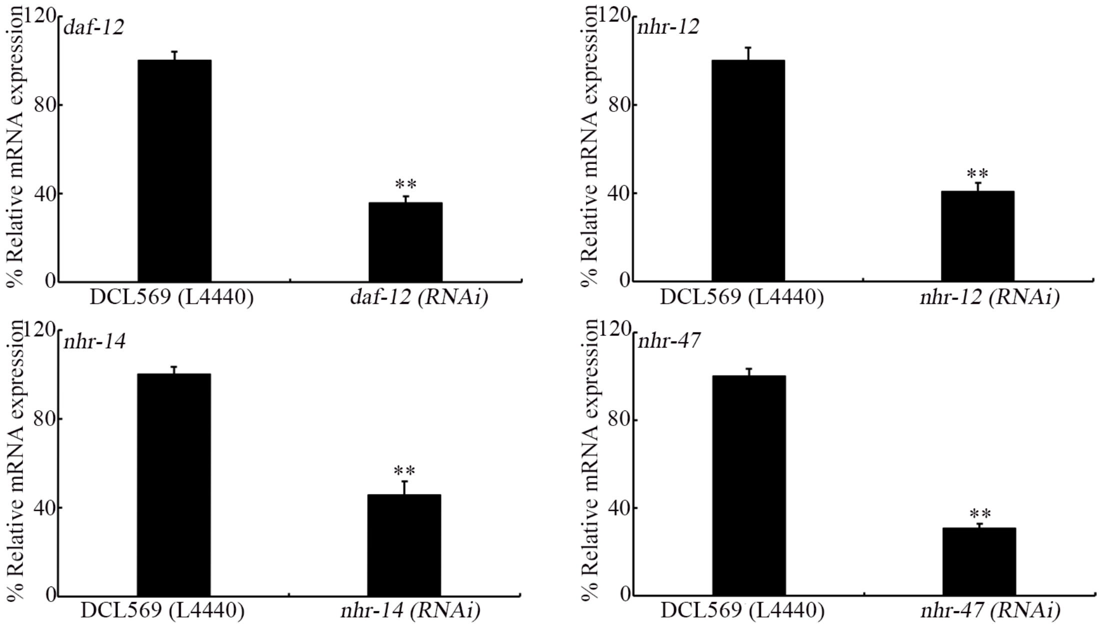

Figure S1. RNAi efficiency of *daf-12, nhr-12, nhr-14* and *nhr-47*. $^{**}P < 0.01$ *vs* DCL569(L4440).

Table S1. Primer information for qRT-PCR.

Gene	Forward primer (5′-3′)	Reverse primer (5′-3′)
nhr-2	CAGTGGCTTCGCCCAATTTT	GTATTACGAATGCCGCCACG
nhr-4	ATTGTCCGGTGGCTGTTGAT	CTCCTTCAGGAAGATGGGCG
nhr-12	TGGAACGGAAACACCGACTA	AGCTGAAGCCTGTGGAACAA
nhr-13	CCCACACCATTGACAGGCTA	CTGACCACGTGGCGAATGTT
nhr-14	TTCTTGCACCCAGAAGCCAA	CCCTGAAATCATTGGCCGTT
nhr-17	ACGACTCTTCATCCACACGG	GTTGGGAGCCGTGTTTTGTC
nhr-20	GAGGCGATGTGCCATTTGAC	GCTGCAAATTCCACGTGTGT
nhr-21	CCAAGTGACAAACGACAGCA	AACCGAGATGCACTTTTTGC
nhr-22	AGAACTGGTGCAAGCGAGAA	ACAGTGCATACGGTCACCAG
nhr-23	CGCGACAACAAGCTCACAAT	GGATGATTGACTGCGTCGGA
nhr-24	ATTGGCGGACGAAATGCTTG	ACAGGCAACGGAAGTGTTGA
nhr-25	TGGTTCTGCGACCGAATCAT	TGTGCTCACTCTCATTGCGT
nhr-34	AGGAGAAGAGTTCACCGGACT	AGGCGAAGTGCTTCCTTCAT
nhr-47	ATGATGAGAGCCCCGAGAGT	TCAGCGTACGGTAGGGATCT
nhr-49	TAGATCCTCTTGCGGAACCA	TGAATGGAGTCCCGTTGAGA
nhr-60	CTAGTGAAACGAGCGCAGGA	GGGGATGGTGGTTTCGGTAG
nhr-61	TGAGTATGTCGACTATAGTGGTG	ACGTGGTTAAAGTGCGAACAA
nhr-66	T	TCGTTCTTGGCTGCAACAAT
nhr-67	ACTTATTACCAGCGGTGGGC	TCCGGCGAATTAGGAGAGGA
nhr-71	CGCCAAGAATGATGACTGCG	ACCGGGGAACAAATCAACACT
nhr-78	ACAGCTCGCCAGAAGTCATC	TCCAGGGACTTGAACAACGG
nhr-114	ATTCGACTTCCGCTCGTACC	TAACGCGAAGCTCCGAAAGA
nhr-145	CTCACAATTTTGGGAGGCACC	GGCATCGGGGTTTGAAAGAC
nhr-147	GGCGTACGAATGCCTTAACC	GGCATCGGGGTTTGAAAGAC
nhr-149	GGCGTACGAATGCCTTAACC	GCTGCATTGATCCATCTGGT
nhr-155	GAGGAGCCATGTCCAGTTTC	GCAATGGTCGAGTCGTGGTA
nhr-158	TCGAGACCTTTCCGATGTGC	TTTGCTGGGCGTTTGGATTG

nhr-171	ACATCAGCCCACAGGATTGG	ACGTTGTGTAGTTCGACGGG
nhr-181	TTACCCAGCGACGGATTGAC	TCCTTCGTCAGCCCTTTGTG
nhr-207	TTCCTGTCGAGCCCACATTC	GTACCCGGCTTGGGAATCTT
nhr-213	CAACCACATGGGAAGGGAGT	CTCTCTGCAGTATCCACCGG
nhr-249	ACGTCTCCAGCGACAATTGT	CATCCCTTTTCTGCCAAATG
daf-12	GGATTATGGGCGGAAATACA	TCTGACGTCGTCGACTCTCT
ins-3	GCAACGGGCGCCAATTAAA	GATGAACATTCTCCTCCA
ins-39	CATAACATTCACTCCCTG	CCCGACGTGTTTGATGGT
daf-28	TTGCAGCCGACGAGTTTC	GCTCATCGCCATCTTTGC
lin-44	GCTCATCGCCATCTTTGC	AGCCGATCACAATCACCTTG
egl-17	ACCCTTGAGCACATTACCGA	TTTGGGGGAGTTGAGCAGAC
efn-3	GGTTTAATGGAAGCGACGCC	CCATACCGTCAGGGATTGGG
lag-2	TTCCCGGTTGCGTTCAAAAC	GGCGTCTTTGACACTGCAAG
daf-2	GACATCGGATGGATGGGACC	TCGCTGGCGACTATGTGA
mig-1	GCTTACGCGATGAGCTGTGAT	TCGGTGCACGAGATTTGGAA
vab-1	CGTCTCGAGATGCTCCACTC	CAACTCCGAACGACCAGACA
	ATGTGCACAGAGATCTCGCC	
tba-1	TCAACACTGCCATCGCCGCC	TCCAAGCGAGACCAGGCTTCAG
